# Methylation-associated down-regulation of *RASSF1A *and up-regulation of *RASSF1C *in pancreatic endocrine tumors

**DOI:** 10.1186/1471-2407-11-351

**Published:** 2011-08-12

**Authors:** Giorgio Malpeli, Eliana Amato, Mario Dandrea, Caterina Fumagalli, Valentina Debattisti, Letizia Boninsegna, Giuseppe Pelosi, Massimo Falconi, Aldo Scarpa

**Affiliations:** 1 Department of Pathology and Diagnostics, University of Verona, Verona, Italy; 2 ARC-NET Center for Applied Research on Cancer, the Hospital and University of Verona, Verona, Italy; 3 Department of Pathology and Laboratory Medicine, National Cancer Institute and University of Milan, Milan, Italy; 4 Department of Surgery and Oncology, the Hospital and University of Verona, Verona, Italy

## Abstract

**Background:**

*RASSF1A *gene silencing by DNA methylation has been suggested as a major event in pancreatic endocrine tumor (PET) but *RASSF1A *expression has never been studied. The *RASSF1 *locus contains two CpG islands (*A *and *C*) and generates seven transcripts (*RASSF1A*-*RASSF1G*) by differential promoter usage and alternative splicing.

**Methods:**

We studied 20 primary PETs, their matched normal pancreas and three PET cell lines for the (i) methylation status of the *RASSF1 *CpG islands using methylation-specific PCR and pyrosequencing and (ii) expression of *RASSF1 *isoforms by quantitative RT-PCR in 13 cases. CpG island A methylation was evaluated by methylation-specific PCR (MSP) and by quantitative methylation-specific PCR (qMSP); pyrosequencing was applied to quantify the methylation of 51 CpGs also encompassing those explored by MSP and qMSP approaches.

**Results:**

MSP detected methylation in 16/20 (80%) PETs and 13/20 (65%) normal pancreas. At qMSP, 11/20 PETs (55%) and 9/20 (45%) normals were methylated in at least 20% of *RASSF1A *alleles.

Pyrosequencing showed variable distribution and levels of methylation within and among samples, with PETs having average methylation higher than normals in 15/20 (75%) cases (*P *= 0.01). The evaluation of mRNA expression of *RASSF1 *variants showed that: i) *RASSF1A *was always expressed in PET and normal tissues, but it was, on average, expressed 6.8 times less in PET (*P *= 0.003); ii) *RASSF1A *methylation inversely correlated with its expression; iii) *RASSF1 *isoforms were rarely found, except for *RASSF1B *that was always expressed and *RASSF1C *whose expression was 11.4 times higher in PET than in normal tissue (*P *= 0.001). A correlation between *RASSF1A *expression and gene methylation was found in two of the three PET cell lines, which also showed a significant increase in *RASSF1A *expression upon demethylating treatment.

**Conclusions:**

*RASSF1A *gene methylation in PET is higher than normal pancreas in no more than 75% of cases and as such it cannot be considered a marker for this neoplasm. *RASSF1A *is always expressed in PET and normal pancreas and its levels are inversely correlated with gene methylation. Isoform *RASSF1C *is overexpressed in PET and the recent demonstration of its involvement in the regulation of the Wnt pathway points to a potential pathogenetic role in tumor development.

## Background

Pancreatic endocrine tumors (PET) are rare neoplasms whose molecular pathogenesis is largely unknown. Silencing of the *RASSF1A *gene by methylation has been proposed as a crucial pathogenetic event in PET by five studies, all of which used the very same methylation- specific PCR (MSP) assay to interrogate the same region of the gene [1-5]. Two of these five papers assessed the methylation status of several candidate tumor suppressor genes and reported the methylation of *RASSF1A *in 75% [3] and 83% [2] of PET. This high rate of *RASSF1A *methylation in PETs was confirmed in the other three studies, where the rate reported ranged from 60% to 100% of cases [1,4,5]. However, the formal proof that the *RASSF1A *gene silencing by methylation in PET is associated with loss of its expression has never been reported.

In tumor types other than PET, *RASSF1A *involvement was assessed by a quantitative MSP assay (qMSP), analyzing a region of *RASSF1A *different from the one investigated by MSP in PET [6-10]. For some of these tumors methylation was associated with down-regulation of the gene expression [11-19].

Ras Association Domain Family 1 (*RASSF1*) is a putative tumor suppressor gene localized at chromosome 3p21.3 that has been reported to inhibit tumor growth in *in vitro *and *in vivo *systems [20-23]. *RASSF1 *locus generates seven different transcript variants (*RASSF1A-G*) by differential promoter usage and alternative splicing [24]. *RASSF1A *has been considered a player of inhibitory functions of Ras on cell growth by acting as downstream agent and as such its inactivation is believed to result in the indirect activation of the Ras pathway. Other studies however have also assigned complex roles in cell functions to this gene [25,26].

Two CpG islands are associated with *RASSF1 *regulatory region: CpG island A extending in the regulatory region common to *RASSF1A, D, E, F *and *G*; CpG island C in the regulatory region of *RASSF1C*. Little is known about the influence of CpG islands on *RASSF1B*. The main *RASSF1 *variants that are ubiquitously expressed in normal tissues are *RASSF1A *and *RASSF1C *[24]. No information is available regarding the expression of *RASSF1 *isoforms in PET.

In this study, we evaluated the putative role of *RASSF1A *methylation as a possible tumor specific and transcription regulatory event occurring in PET. To this end, we applied both the MSP and qMSP approaches indicated above in a set of 20 PETs and matched normal pancreas together with the evaluation of *RASSF1 *gene expression in 13 cases. We also assessed the methylation status of each of the CpG sites encompassing a large area of the regulatory region, including the CpGs interrogated by MSP and qMSP assays, with methylation-sensitive pyrosequencing. This technique allows relative quantification of methylated CpGs in relation to unmethylated CpGs for each of the CpG sites tested.

## Methods

### Samples and nucleic acids extraction

Twenty primary sporadic PET (Table 1) with matched normal pancreas were studied. Samples were obtained in accordance with the Verona University and Hospital Trust Ethics Committee and PET were classified according to WHO criteria [27]. Normal pancreas was taken far away (at least 2 cm) from the neoplastic lesion and was macroscopically and histologically normal at pathological evaluation. All PET samples were negative for *MEN1 *gene mutation (data not shown) and there was no family history for *MEN1, VHL *or any other familial cancer predisposing syndrome.

**Table 1:** Clinical data of PET cases

Case	Sex	Age (years)	Tumor type	WHO classification*
1	M	53	non-functioning	WDEC
2	M	42	non-functioning	WDEC
3	M	51	insulinoma	WDET
4	M	44	non-functioning	WDEC
5	M	48	non-functioning	WDEC
6	M	51	insulinoma	WDET
7	F	66	non-functioning	WDET
8	F	65	non-functioning	WDET
9	M	35	non-functioning	WDEC
10	F	70	non-functioning	WDEC
11	M	76	non-functioning	WDEC
12	F	40	non-functioning	WDEC
13	M	41	insulinoma	WDET
14	M	40	non-functioning	WDEC
15	M	68	non-functioning	WDEC
16	F	47	non-functioning	WDET
17	M	48	non-functioning	WDET
18	M	41	gastrinoma	WDEC
19	M	70	non-functioning	WDEC
20	F	46	gastrinoma	WDET

* WDEC: well-differentiated endocrine carcinoma; WDET: well-differentiated endocrine tumor.

Nucleic acids were prepared from 12 cryostatic sections, with tumor cellularity checked every four sections. Tumor cellularity was always over 90%. DNA was purified by QiAamp DNA Mini Kit (Qiagen), following manufacturer's instructions. RNA was extracted with TRIzol Reagent (Invitrogen) following the manufacturer instructions, then treated with DNAse I (Invitrogen) for 15 minutes at room temperature, and finally incubated at 65°C for 10 minutes for enzyme inactivation. As a control of the DNAse I efficiency, a DNA sample was subjected to DNAse I treatment at the same conditions and loaded on agarose gel.

### Cell lines and demethylating treatment

PET cell lines CM, QGP1 and BON were grown in culture medium containing RPMI 1640 supplemented with 2 mM glutamine and 10% FBS and incubated at standard conditions (37°C with 5% CO_2_). Cell lines were treated with the enzymatic inhibitor 5'-aza-2'-deoxycytidine (DAC) (Sigma-Aldrich), previously solubilized in DMSO and stored at -80°C until use. Cells treated with the inhibitor were grown in presence of 2.5 μM DAC for 6 days.

### Optimization of bisulfite treatment of DNA

DNA was chemically modified with sodium bisulfite using MethylSeq Kit (Applied Biosystems) to convert unmethylated cytosine to uracil, while methylated cytosines resist conversion. Duration of bisulfite treatment necessary to determine complete cytosines modification was assessed by incubating DNA with sodium bisulfite for 4, 8 and 16 hours at 50°C in a heat-block. Complete conversion of cytosines occurred after 16 hours treatment and the DNA obtained was used for subsequent experimental procedures.

### RASSF1A methylation-specific PCR (MSP)

Methylation-specific PCR (MSP) was performed according to Pizzi et al. [5]. Reference unmethylated DNA was from healthy donor peripheral blood mononuclear cells. Reference full methylated DNA was CpGenome Universal Methylated DNA (Chemicon International).

### Quantitative methylation-specific PCR

Quantitative MSP (qMSP) was performed as described [6,8] with minor modifications reported in Additional file 1. Primers are listed in Additional file 2, Table S1. The methylation level of *RASSF1A *was calculated as the ratio of methylated *RASSF1A *to *MYOD1*, the latter representing the total input unmethylated DNA [28]. To classify samples as methylated, we applied the same cut-off used by Xing et al. [10]. The 20% cut-off point at qMSP analysis was chosen with a conservative assumption that all of the cells in a sample were tumor cells without normal cell contamination and without loss of heterozygosity in the *RASSF1A *locus. Thus, a cut-off point of 20% of total alleles for *RASSF1A *methylation implies that 20% of all of the tumor cells carry *RASSF1A *methylation, if both alleles are methylated in one cell. In reality, the tumor tissue is not 100% pure, and there may be also loss of heterozygosity in the *RASSF1A *locus. Therefore the 20% cut-off point of total alleles for *RASSF1A *methylation actually reflects the presence of a clone of more than 20% of the total number of tumor cells [10].

### Pyrosequencing of bisulfite-modified DNA

Bisulfite-modified DNAs were evaluated by pyrosequencing [29] as recommended (Biotage AB), using primers and conditions as reported in Additional file 1 and in Additional file 2, Table S1. The degree of methylation at each CpG position was determined from the ratio of C and T by the Pyro Q-CpG Software (Biotage AB). Pyrosequencing was performed on the sense and antisense strand of *RASSF1A *(nucleotides -163 to +262 in chromosome 3: 50353109-50353534, NC_0000003.10) and of *RASSF1C *(nucleotides -86 to +193 in chromosome 3: 50349706-50349985, NC_0000003.10).

### Identification of alternatively spliced mRNA isoforms of RASSF1 transcribed from CpG island A

Isoforms *A, D, E, F *and *G *originate from alternative splicing of *RASSF1A *gene. Taking advantage of their different length, isoforms *A, D, E *and *F *were identified by microfluidic chip-electrophoretic separation (DNA 1000 chip, 2100 Bioanalyzer, Agilent Technologies) of PCR products. The PCR amplification of cDNAs used primers designed in the first and last exon of the gene (NCBI Reference Sequence NM_007182). As *RASSF1G *lacks the last exon of *RASSF1A*, it was amplified separately with opportune primers. The PCR primers used are listed in Additional file 2, Table S1.

### Measurement of mRNA expression by quantitative reverse transcription-polymerase chain reaction (qRT-PCR)

RNA samples were retrotranscribed to cDNA using the First Strand cDNA Synthesis Kit (Roche). A reverse transcriptase minus cDNA was prepared for each sample as a control. qRT-PCR conditions and primers are reported in Additional file 1 and in Additional file 2, Table S1. The relative expression level was calculated using transcript level of *RPLPO *as reference gene and the standard (= 1) was the average of the levels of expression of all samples. qRT-PCR data analysis was performed according to the comparative method following the User Bulletin #2 (Applied Biosystems).

### Statistical analysis

Pearson's correlation (r) and Student's *t-test *were used to compare mRNA expression and differentially methylated regions between groups of samples. Pairwise *t-test *or Spearman Rank (rho) correlation was used to compare expression in matched PET and normal samples. *P *values less than 0.05 were considered statistically significant.

## Results

**Figure 1:**
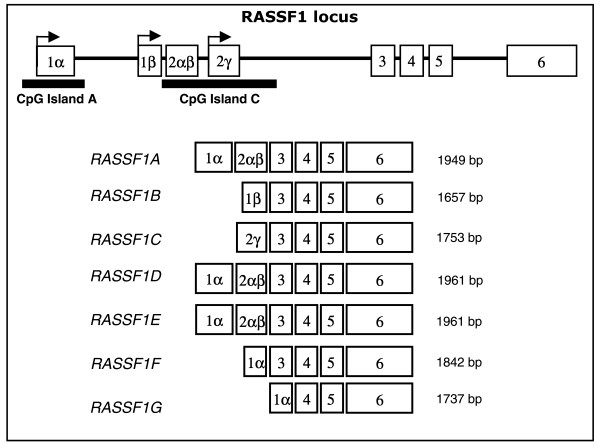
**Schematic representation of the *RASSF1 *locus on chromosome 3p21.3 and its transcription map**. White boxes represent the exons and the bold line represents the introns. *RASSF1A, RASSF1B *and *RASSF1C *variants are generated by differential promoter usage (arrows). *RASSF1D, E, F, G *are variants derived from alternative splicings of *RASSF1A*. Two CpG islands (black bands below the sequence) are associated to *RASSF1 *promoter region: CpG island A (737 bp, 85 CpGs) extending in the promoter region of *RASSF1A, D, E, F *and *G*; CpG island C (1365 bp, 139 CpGs) in the regulatory region of *RASSF1B *and *C*.

The methylation status of CpG islands A and C of the *RASSF1 *locus (Figure 1) was assessed in a series of 20 PET and matched normal tissues (Table 1). Methylation of CpG island A was first evaluated by the qualitative methylation-specific PCR (MSP) used in papers dealing with PET [1-5] and then by a quantitative methylation - specific PCR (qMSP) applied in the study of other tumor types [6-10]. The regions of CpG island A analyzed by MSP and qMSP were further investigated by pyrosequencing, which provided the methylation status of 51 CpGs of the island A that also encompasses those explored by MSP and qMSP approaches. Analysis of the expression of the diverse *RASSF1 *isoforms completed the study. The analysis of CpG island C was performed by pyrosequencing analysis of 37 individual CpGs.

### Methylation-specific PCR showed a high frequency of RASSF1A methylation in PET and normal pancreas

MSP detected methylation in 16 of 20 (80%) PETs and 13 of 20 (65%) normal pancreas (Additional file 3, Figure S1A). In particular, methylation was detected in 12 PETs and matched normals, was absent in both PET and matched normal in 3 cases, while in five cases it was discordantly present in either PET (4 cases) or normal (1 case).

### Quantitative methylation-specific PCR showed variable RASSF1A methylation among PET and normal pancreas

All PET and normal samples showed variable levels of methylation at qMSP, but only 11/20 PETs (55%) and 9/20 (45%) normals had a methylation level in at least 20% of the *RASSF1A *alleles (Additional file 3, Figure S1B). The cut-off point of 20% of total alleles for *RASSF1A *methylation implies that 20% of cells in a sample carry *RASSF1A *methylation [10].

Using this cut-off, methylation in paired PET/normal cases was as follows: 6 both methylated, 6 both unmethylated, 5 methylated in PET, 3 methylated in normal. The qMSP methylation levels in PET and matched normals showed a higher degree of methylation in neoplastic tissues with respect to normals in 8 cases and a lower degree in 9 cases; the remaining 3 cases had similar methylation levels in PET and normal.

### Pyrosequencing revealed that RASSF1A promoter and first exon are variably methylated within and among PETs and normal pancreas

**Figure 2:**
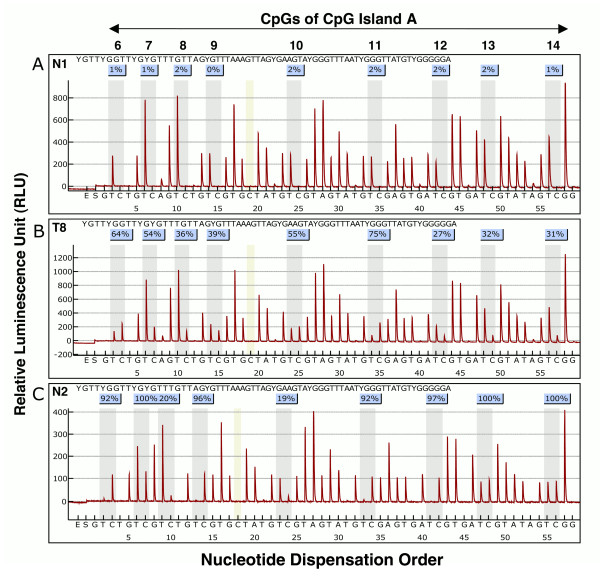
**Analysis of DNA methylation by pyrosequencing**. Representative results of pyrosequencing showing analysis of 9 CpGs in the first exon of *RASSF1A *(from CpG6 to CpG14). The y-axis represents the signal intensity of luminescence in relative luminescence units (RLU) emitted following nucleotide base incorporation into the sequence, while the x-axis shows the nucleotide dispensation order, indicated by the letters below each graph: E, enzyme mix; S, substrate; A, G, C, T, nucleotides. The sequence in the upper left of the boxes is the region subjected to pyrosequencing analysis, where the nine letters Y indicate the respective cytosine in the 9 CpG sites (from CpG6 to CpG14) whose methylation status is shown here. Values in light blue boxes are the percentages of methylation of each CpG. Percentages of methylation represent the ratio between signal intensities of C and T in each C of a CpG site. Dispensations corresponding to the potentially methylated cytosine (C or T after bisulfite treatment) are highlighted in grey. Each panel depicts a representative sample showing no methylation (panel A, sample N1), partial methylation (panel B, sample T8) and high methylation (panel C, sample N2) levels.

**Figure 3:**
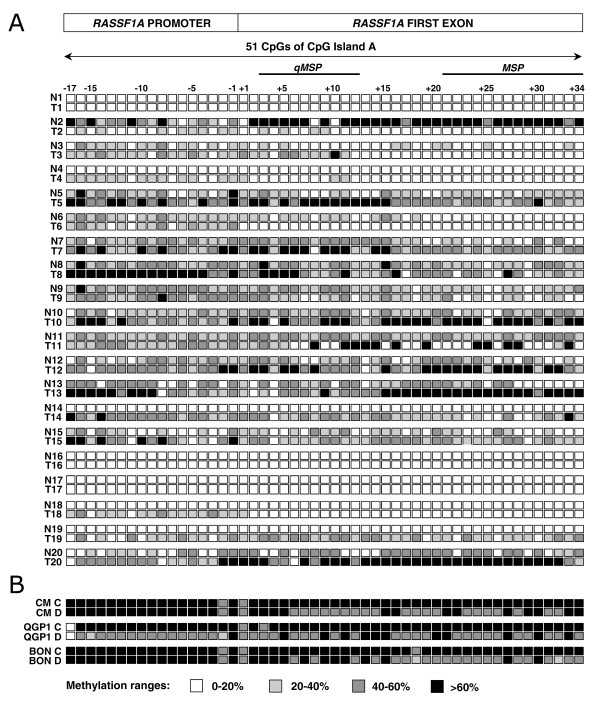
**Methylation status of CpG island A in PET, normal pancreas and cell lines.** Panel A shows the methylation level of the 51 CpGs analyzed by pyrosequencing in the 20 matched normal/tumor samples. Numbers on the left refer to normal (N) and tumor (T) samples of the 20 PET listed in Table 1. Each of the 51 CpGs, 17 in the promoter region and 34 in the first exon, is represented by a square. Numbers on top show the location of CpG dinucleotides and transcription start site is indicated (+1). Any CpG is represented by a square that has one of four grey levels according to the proportion of methylation detected, namely white, light grey, dark grey and black indicating a level of methylation of 0-20%, 20-40%, 40-60% and > 60%, respectively. Black lines on top of squares indicate location of qMSP and MSP assays spanning CpGs 3 to 12 and CpGs 21 to 34, respectively. Panel B reports the methylation status of the 51 CpGs in the three indicated PET cell lines (CM, QGP1 and BON), where C and D indicates untreated control cells and cell lines treated with demethylating agent, respectively.

Pyrosequencing was applied to determine the methylation level of 51 CpGs within CpG island A of *RASSF1A*, including 17 CpGs in the promoter and 34 CpGs in the first exon. In fact, this technique is able to provide the level of methylation of single CpGs (Figure 2). *RASSF1A *methylation showed a high variability in terms of distribution and level among PET and normal samples (Figure 3). Analysis of the *RASSF1A *antisense DNA strand confirmed the results obtained for the sense strand.

Table 2 lists the average methylation levels of promoter, first exon and of the CpG island A of *RASSF1A *in 20 tumors and their matched normal samples. By considering the average methylation of all 51 CpGs, methylation was higher in tumor than in normal tissue in 15/20 (75%) cases (pairwise *t-test, P *= 0.01) (Figure 3, Table 2); of the remaining 5 cases, 2 cases had similar methylation level in normal and tumor (cases 1 and 9), while 3 cases had normal showing higher methylation than tumor (cases 2, 6, 16).

**Table 2:** Pyrosequencing analysis of 20 matched samples: average methylation values of 51 CpGs in CpG island A of *RASSF1A*, of which 17 CpGs in the promoter and 34 CpGs in the first exon

	Average methylation (%)		
Cases*			
	
	Promoter	First Exon	CpG island A
N1	4	2	3
T1	2	4	3
**N2**	**34**	**75**	**60**
**T2**	**20**	**11**	**14**
N3	29	14	19
T3	37	19	25
**N4**	**2**	**2**	**2**
**T4**	**23**	**8**	**13**
N5	37	22	28
T5	59	56	57
**N6**	**27**	**18**	**22**
**T6**	**29**	**10**	**17**
N7	40	35	37
T7	53	52	52
**N8**	**47**	**37**	**40**
**T8**	**77**	**46**	**57**
N9	43	32	35
T9	46	29	35
**N10**	**35**	**34**	**34**
**T10**	**49**	**72**	**64**
N11	37	38	38
T11	38	43	41
**N12**	**28**	**18**	**22**
**T12**	**42**	**66**	**57**
N13	25	34	31
T13	67	65	66
**N14**	**4**	**3**	**4**
**T14**	**49**	**30**	**37**
N15	36	28	31
T15	50	37	41
**N16**	**14**	**8**	**10**
**T16**	**10**	**9**	**9**
N17	3	2	2
T17	3	5	4
**N18**	**4**	**18**	**13**
**T18**	**32**	**7**	**16**
N19	10	6	8
T19	25	37	33
**N20**	**25**	**36**	**32**
**T20**	**43**	**80**	**67**

*N = normal pancreas, T = PET.

By considering a threshold as for qMSP, a sample was defined as "methylated" if it had an average methylation across 51 CpGs higher than 20%. Using this threshold, 13/20 (65%) tumors and 12/20 (60%) normal samples were methylated. Among the 3 normal samples with higher degree of methylation than the neoplastic counterpart, case 2 showed the largest difference of average methylation level between PET and normal.

Pyrosequencing results overlapped those obtained by qMSP in both PET and normal pancreas when the status of the 10 CpGs included in the qMSP assay (from CpG3 to CpG12; see Figure 3) was considered; in fact, the average methylation level of these 10 CpGs by pyrosequencing *vs *the level of methylation by qMSP showed a good correlation (r = 0.78, *P *= 0.0001).

### RASSF1A is methylated in normal pancreas

Regardless of technique used, our data indicate that *RASSF1A *methylation occurs in normal pancreas, as previously reported by others [2,4,5,30], and presents inter-individual variability in terms of frequency and level.

### RASSF1A mRNA expression in PET is lower than that of matched normal pancreas and inversely correlates with methylation level of CpG island A

**Figure 4:**
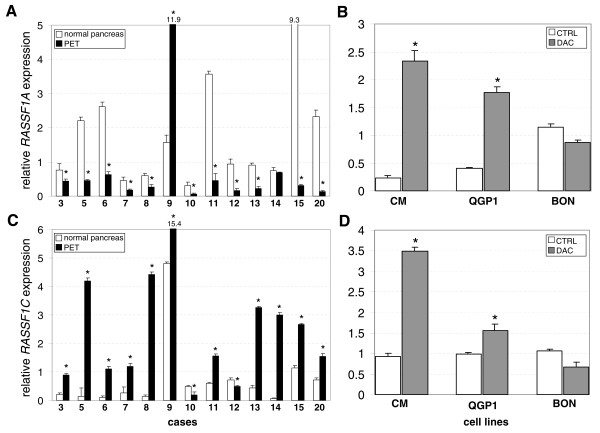
**Expression of *RASSF1A *and *RASSF1C *in PET, normal pancreas and PET cell lines**. The expression levels of *RASSF1A *(A) and of *RASSF1C *(C) in 13 PET (black bars) and matched normal pancreas (white bars) are shown. Expression data are the mean of three measures obtained by quantitative RT-PCR. Data were normalized using the expression level of the gene *RPLPO *as an internal reference. Data were analyzed according to the comparative method and standard (= 1) was represented by the average expression of all samples. Numbers under the bars refer to PET cases listed in Table 1. Expression level of *RASSF1A *(B) and of *RASSF1C *(D) (mean ± SD) in PET cell lines QGP1, BON, CM are represented by white bars for control untreated (CTRL) and dark grey bars for 5'-aza-2'-deoxycytidine (DAC) treated cells. In (A) and (C) an asterisk indicates a significant difference of expression of *RASSF1A *and *RASSF1C *in normal with respect to PET (*t-test, P *< 0.05) (see Additional file 2, Table S2 for expression data and statistics). In (B) and (D) an asterisk indicates a significant difference of expression in treated cells with respect to the same cells untreated (*t-test, P *< 0.05).

Expression of *RASSF1A *mRNA was assessed in 13 PETs and matched normal pancreas by qRT-PCR. Expression was lower in PET than in normal in 11 of 13 cases, equal to an average difference of 6.8 fold (*P *= 0.003) (Figure 4A and Additional file 2, Table S2).

**Figure 5:**
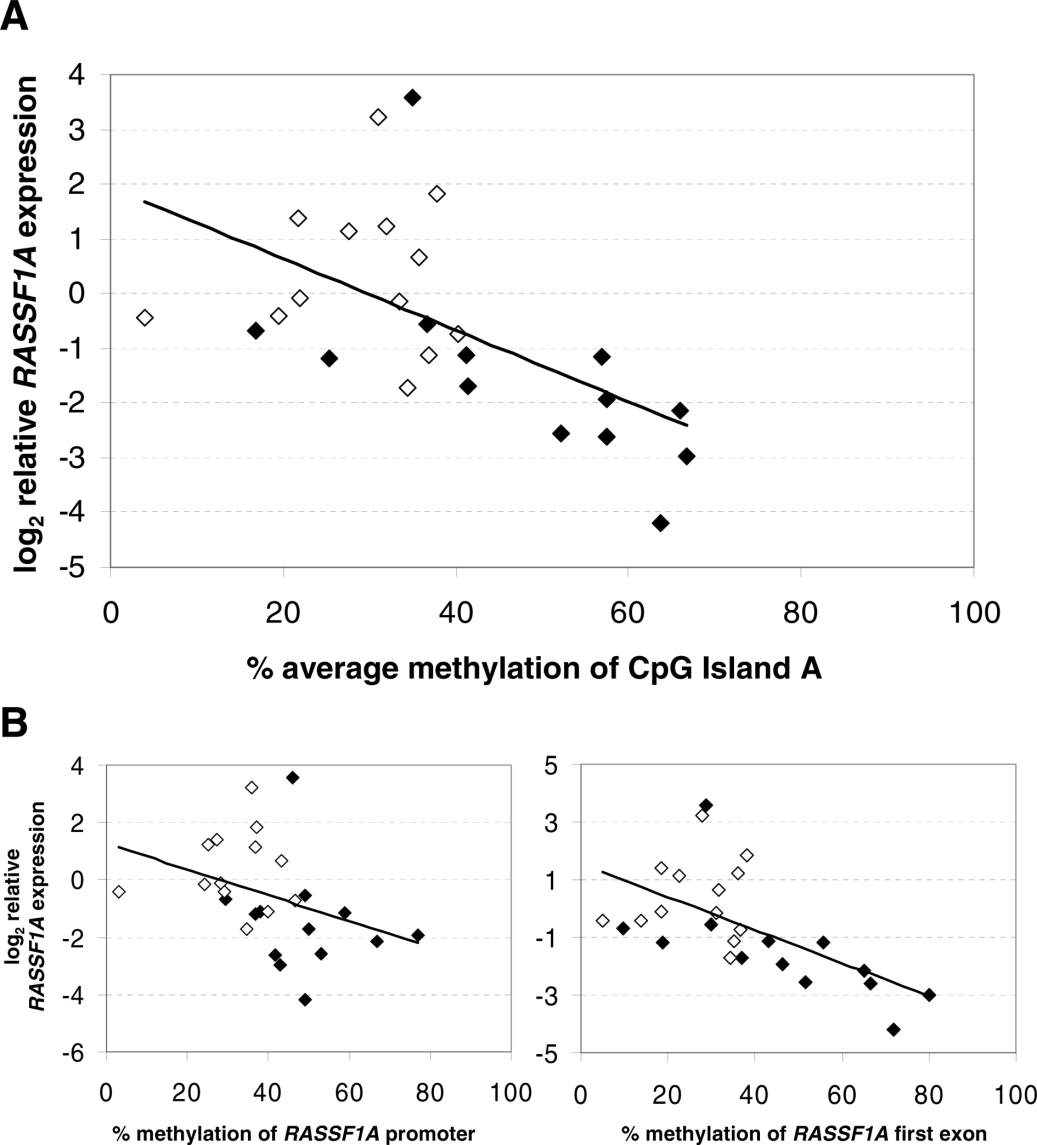
**Relationship between the expression of *RASSF1A *and methylation of CpG island A**. (A) Expression of *RASSF1A *in function of the average methylation of 51 CpGs in the CpG island A in 13 PET (black diamond) and matched normal pancreas (white diamond). A linear regression through the data (y = -0.0673x + 2.0228, R^2 ^= 0.337) describes the relationship between methylation and gene expression. Only tumor samples had an average methylation > 40% while all normal tissues had an average methylation < 40%. (B) *RASSF1A *gene expression *vs *average methylation of 17 CpGs in the promoter (graph on the left) and *vs *that of 34 CpGs in the first exon (graph on the right) in PET cases (black diamond) and normal pancreas (white diamond). A linear regression through the data describes the relationship between methylation of promoter (y = -0.0452x + 1.2697, R^2 ^= 0.1314) and first exon (y = -0.0572x + 1.5418, R^2 ^= 0.3569) and gene expression.

The comparison between mRNA expression levels and methylation status by MSP showed that the two events were not significantly associated (Wilcoxon test, *P*= 0.07). When comparing *RASSF1A *expression data with percent of methylation by qMSP, Spearman's test revealed the presence of an inverse correlation (r=-0.35, *P *= 0.08), which became significant when the average methylation of the 51 CpGs of pyrosequencing data was considered (r=-0.57, *P *= 0.01) (Figure 5A). A major contribution to this latter association was provided by the first exon (r=-0.61, *P *= 0.007) while methylation of promoter did not correlate significantly (r=-0.44, *P *> 0.05) (Figure 5B).

We then searched for a role of specific CpGs in the transcription regulation of *RASSF1A *using pyrosequencing data (Additional file 3, Figure S2). The highest degree of correlation was found for CpG5 (inner graph in Additional file 3, Figure S2). The expression of *RASSF1A *inversely correlated with the methylation of this CpG (r=-0.78, *P *= 0.001).

### RASSF1A expression is present in PET cell lines despite a strong methylation in CpG island A and is enhanced by treatment with demethylating agents

The three PET cell lines CM, QGP1, BON showed a level of methylation above 90% by qMSP (Additional file 3, Figure S1B). Pyrosequencing analysis confirmed the high level of methylation throughout the CpG island A (Figure 3B). Interestingly, *RASSF1A *was always expressed at similar level in all the untreated cell lines (Figure 4B). *RASSF1A *was always expressed in all the untreated cell lines, but at higher level in BON compared to QGP1 and CM.

In order to evaluate the effect of methylation on gene expression of *RASSF1A*, the three cell lines CM, QGP1 and BON were treated with the demethylating agent 5'-aza-2'-deoxycytidine (DAC). Treatment reduced methylation by about 25% from the starting level. The main changes of methylation regarded the CpGs included in the first exon (Figure 3B). However, DAC treatment induced a significant increase of *RASSF1A *expression in QGP1 and CM, but not in BON cell line (Figure 4B). The mRNA expression levels and the average methylation of the 51 CpGs within CpG island A showed a significant inverse correlation (r=-0.73, *P *= 0.004).

### Isoform RASSF1C is highly expressed in PET at variance with the other six isoforms generated from RASSF1 locus

Seven mRNA isoforms could potentially be expressed by the *RASSF1 *locus and four of them (*RASSF1-A, D, E *and *F*) are generated from differential splicing of *RASSF1A *(Figure 1).

**Figure 6:**
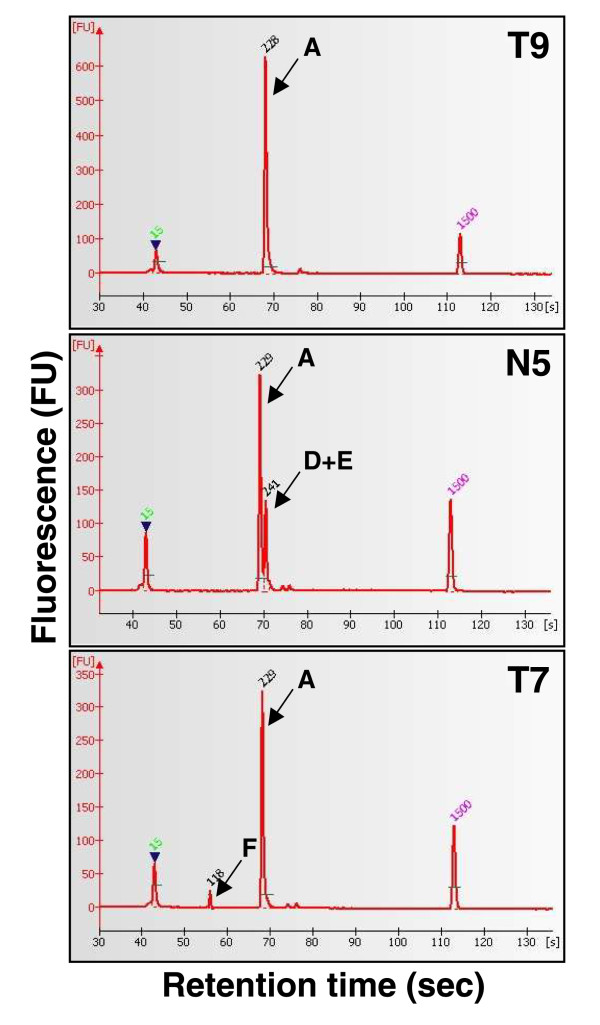
**Identification of *RASSF1A *and its splicing variants by PCR**. Electropherograms of PCR products obtained by amplification of cDNA. PCR products were loaded on microfluidic DNA 1000 chip and run on a 2100 Bioanalyzer instrument (Agilent Technologies). The amplicon of isoform A is 229 bp, both isoforms *D *and *E *generate a 241 bp peak, due to the insertion of 12 bp to the sequence of isoform A, and therefore cannot be separated, while the amplicon of isoform *F *is the shortest one (118 bp). Peaks at 15bp and 1500 bp are the lower and upper size markers, respectively. Case T9 expresses isoform *A*, N5 expresses isoform *A *and *D*/*E*, T7 expresses isoform *A *and *F*.

PCR products of *RASSF1 *isoforms *A, D, E *and *F *are resolved by microfluidic chip electrophoresis (Figure 6). *RASSF1A *was detected in all PETs while the splicing variants *D, E *and *F *were rarely seen: *RASSF1F *was found in one PET (T7) and two normal pancreas (N3, N5), *RASSF1D*/*RASSF1E *was expressed in only one sample (N5).

Of the remaining three isoforms, *RASSF1G *was always absent in PET and normal pancreas, while variant *B *was always expressed in both normal and PET with no significant difference (data not shown). Conversely, *RASSF1C *expression level in PETs was significantly higher than that found in normals in 11 of 13 cases (*P *= 0.001) (Figure 4C), having a fold average expression ratio PET/normal of 11.4.

### CpG island C is never methylated

Pyrosequencing analysis revealed that none of the 37 CpGs of CpG island C is methylated in PETs and normal pancreas.

### Lower expression of RASSF1A and higher expression of RASSF1C in PET with respect to normal pancreas are concomitant events

We tested the relationship between *RASSF1A *and *RASSF1C *analyzing the expression ratio PET/normal. Comparison of *RASSF1A *PET/normal and *RASSF1C *PET/normal expression ratios suggested that the lower expression of the first and the higher expression of the second are two events occurring concomitantly (r = 0.7, *P *= 0.01).

### CpG island C is never methylated in PET cell lines and treatment with demethylating agents enhances mRNA expression of RASSF1C

No methylation was detected in 37 CpGs of CpG island C in PET cell lines using pyrosequencing and *RASSF1C *transcript was always found at similar level in untreated cells (Figure 4D). However, treatment of cell lines with DAC upregulated *RASSF1C *expression significantly in QGP1 and CM, but not in BON cell line.

## Discussion

The suggestion that *RASSF1A *silencing by methylation is involved in the pathogenesis of PET has been supported by the presence of methylation in the CpG island A of the gene [1-5]. However, all studies used the same qualitative method to assess methylation and *RASSF1A *expression has never been analyzed in PET.

In this study we conducted an exhaustive analysis of *RASSF1 *methylation status in order to define its putative role as a possible tumor specific and transcription regulatory event occurring in PET.

We first analyzed *RASSF1 *methylation by the qualitative MSP assay because this was the method used in previous studies on PET, and found methylation in 80% of cases, a frequency similar to that reported in those studies [1-5]. However, MSP is highly sensitive recognizing as little as 0.1% of methylated alleles [31], thus classifying a sample as methylated on the basis of a minimal proportion of methylated target. We then performed qMSP that has been previously used for the evaluation of methylation status of *RASSF1A *in tumors other than pancreatic [6-8,10]. This assay analyzes a region of CpG island A that has never been investigated in PET. By this quantitative assay, all PET showed some degree of methylation but only 55% of cases had at least 20% of their alleles methylated.

In normal pancreas, *RASSF1 *methylation was found in 65% of cases by MSP, a value that fit within the range of published studies that used the same technique [2,4,5]. By qMSP assay, 45% of normals had at least 20% of their alleles methylated.

Finally, we obtained a detailed mapping of *RASSF1A *methylation by pyrosequencing, assessing the level of methylation of each of 51 CpGs within CpG island A, also encompassing those explored by MSP and qMSP approaches. Pyrosequencing provided a portrait of the complexity of the methylation pattern of tumor cells, where *RASSF1A *methylation showed a high variability in terms of distribution and level within and among samples. Similar to pancreas, a very complex distribution of methylation of *RASSF1A *was found in breast cancer [19].

Our pyrosequencing data showed that most of normal and tumor samples had an average methylation levels of the CpG island A below 40%, with the exception of one normal (case 2) and seven tumors, having values above 40% (Table 2). To classify samples as "methylated", we applied the same cut-off used for qMSP (an average methylation level higher than 20%). Methylated samples were 13/20 (65%) in PET and 12/20 (60%) in normal pancreas. In matched samples, the average methylation level of the CpG island A was higher in PET than in normal samples in 15/20 (75%) cases (*P *= 0.01). Among the 3 normal samples with higher degree of methylation than the neoplastic counterpart, case 2 showed the largest difference of average methylation level between PET and normal. We repeated the pyrosequencing analysis of this case with same results and did not find any particular features in the clinical profile of the patient to associate to the abnormal methylation data.

Although the overall CpG island A methylation level revealed by pyrosequencing was higher in PET than in normal tissue (Figure 3), normal pancreas displayed considerable methylation levels. The common occurrence of methylation of *RASSF1A *in normal pancreas suggests that this epigenetic event might represent a "field defect", consisting in widespread epigenetic changes arising early in the pancreas before tumor onset, a hypothesis previously suggested [32,33].

When comparing the three techniques employed in this study to investigate the methylation status, MSP showed different results from those obtained by both qMSP and pyrosequencing. This is consistent with the qualitative (MSP) and quantitative (qMSP and pyrosequencing) nature of these approaches. Conversely, qMSP and pyrosequencing gave comparable results (correlation r = 0.78), thus supporting the choice of using the same cut-off for both quantitative results to classify a sample as methylated.

Whatever the method used to detect methylation, the majority of cases showed concordantly methylated or unmethylated tumor/normal pairs, and, when discordant, the methylation was higher in tumor in most cases, except case 2 (Table 2). This raises the question of whether methylation affects the expression of *RASSF1 *gene. Indeed, none of the previous papers suggesting the inactivation of *RASSF1A *in PET due to hypermethylation analyzed gene expression [2,4,5]. In the present work, we evaluated the mRNA expression of *RASSF1 *variants and showed that: i) all PETs and their matched normal tissues expressed *RASSF1A*; ii) the average expression of *RASSF1A *in PET was 6.8 times lower than that in normal tissues (see Figure 4A and Additional file 2, Table S2); iii) the overall extent of *RASSF1A *methylation in PET correlated inversely with its expression and the role of methylation of the first exon seems more important than that of the promoter region (see Figure 5). Accordingly, a correlation between *RASSF1A *expression and the average methylation of the 51 CpGs of island A was found in two of the three PET cell lines analyzed. In all untreated cell lines *RASSF1A *was always expressed despite a strong methylation in CpG island A; in particular, BON cell line showed a higher level of *RASSF1A *expression compared to QGP1 and CM. Moreover, the treatment with the demethylating agent 5'-aza-2'-deoxycytidine enhanced significantly *RASSF1A *expression in QGP1 and CM, but not in BON. This difference in expression levels and response to demethylating treatment is consistent with a possibly different genetic background. Indeed, it has been recently shown that CM, QGP1, and BON harboured different gene mutations; in particular, they had mutations in *FLT1*/*VEGFR1, FGFR3*, and *PIK3CA*, respectively [34].

The expression of *RASSF1A *splicing isoforms *D, E, F *and *G*, and that of the major variants deriving from alternative promoter usage, *RASSF1B *and *RASSF1C*, has never been studied in PET. Here we report that *RASSF1 *isoforms *D, E, F *were rarely expressed in PET and normal pancreas; *RASSF1G *was never found; *RASSF1B *was always expressed in both PET and normal pancreas, with no significant difference; *RASSF1C *expression was averagely 11.4 times higher in PET than in normal tissue. Pyrosequencing analysis revealed that all the CpGs within CpG island C lacked methylation in both tumor and normal tissues. The same situation was found in PET cell lines, where CpG island C was never methylated, and *RASSF1C *was always expressed. Interestingly, treatment with 5'-aza-2'-deoxycytidine enhanced mRNA expression significantly in two of the three cell lines.

The finding of hyperexpression of *RASSF1C *in PET is of great interest in the light of the recently reported role of *RASSF1C *in inhibiting ß-catenin degradation [35]. Thus, *RASSF1C *overexpression may represent a pathogenetic event in PET contributing in sustaining Wnt signaling that has been recently shown to regulate proliferation of pancreatic ß-cells [36]. Moreover, *RASSF1C *has also been implicated in promoting cell migration and attenuating apoptosis in breast cancer cells [37].

## Conclusions

*RASSF1A *gene is frequently methylated in PET and at higher level than in normal pancreas. However, as no more than 75% of cases show *RASSF1A *to be more methylated in PET than normal pancreas, *RASSF1 *gene methylation cannot be considered a marker lesion for this neoplasm.

*RASSF1A *is always expressed in PET and normal pancreas and its levels are inversely correlated with gene methylation status.

Isoform *RASSF1C *is highly expressed in PET at variance with the other six isoforms generated from *RASSF1 *locus; this suggesting that *RASSF1C *might play a pathogenetic role in tumor development, in the light of the recent demonstration of its involvement in the regulation of Wnt pathway.

## Competing interests

The authors declare that they have no competing interests.

## Authors' contributions

GM and EA designed and carried out the experiments, analyzed the results, performed the statistical analysis and wrote the manuscript. MD performed cell cultures and demethylating treatments. VD and CF performed part of the experiments. LB selected the series based on clinical data and tissue availability. GP and MF evaluated critically the data and assisted in manuscript preparation. AS conceived the study and finalized the manuscript. All authors read and approved the final manuscript.

## Pre-publication history

The pre-publication history for this paper can be accessed here:

http://www.biomedcentral.com/1471-2407/11/351/prepub

## Supplementary Material

Additional file 1 **Additional methods**. The file completes the description of methods and experimental conditions employed to obtain the data. The description is subdivided in: quantitative methylation-specific PCR (qMSP), PCR conditions used to amplify samples for DNA pyrosequencing, quantitative reverse-transcription polymerase chain reaction (qRT-PCR) and immunofluorescence procedure. Click here for file

Additional file 2 **Additional tables. Table S1**. Oligonucleotides and experimental conditions used for quantitative MSP (qMSP), DNA pyrosequencing, microfluidic chip-electrophoretic separation (RT-PCR) and quantitative RT-PCR (qRT-PCR). The table lists the primers sequences and the PCR conditions used in qMSP, RT-PCR and qRT-PCR. **Table S2**. Expression and statistics data of *RASSF1A *and *RASSF1C *in PET and normal pancreas obtained by quantitative RT-PCR (qRT-PCR). The table provides the expression data of *RASSF1A *and *RASSF1C *and the statistical analysis. Click here for file

Additional file 3 **Additional figures. Figure S1**. Analysis of methylation of *RASSF1A *by methylation-specific PCR (MSP) and quantitative MSP (qMSP). The figure shows examples of MSP results and a graph representing data obtained by qMSP. **Figure S2**. Pearson's correlations (r) between expression of *RASSF1A *and the average methylation of single CpGs. The graph shows the Pearson's correlation values (r) between *RASSF1A *expression level and the average methylation level for each CpG of the CpG island A. Click here for file
