# Supplementary material for: Methylation-associated down-regulation of RASSF1A and up-regulation of RASSF1C in pancreatic endocrine tumors
Source: BMC Cancer. 2011 Aug 12;11:351. doi: 10.1186/1471-2407-11-351 (PMC3170651; doi:10.1186/1471-2407-11-351)
Supplement: Additional file 1 — Additional methods. The file completes the description of methods and experimental conditions employed to obtain the data. The description is subdivided in: quantitative methylation-specific PCR (qMSP), PCR conditions used to amplify samples for DNA pyrosequencing, quantitative reverse-transcription polymerase chain reaction (qRT-PCR) and immunofluorescence procedure. [file 1471-2407-11-351-S1.PDF]

Additional file 1- Additional methods

Quantitative methylation-specific PCR (qMSP)

PCR amplifications were performed on an ABI Prism 7900HT SDS (Applied Biosystems) in 25 µl total volume containing 10 ng of modified DNA, 67 mM Tris-HCl pH 8.8, 16.6 mM $(NH_4)_2SO_4$, 0.2 mM deoxynucleotides, 2.5 mM $MgCl_2$, 10 mM 2-mercaptoethanol, 1U DNA polymerase Taq Platinum (Invitrogen), 0.8 µM each primers and 0.4 µM probe. Each sample was evaluated in triplicate and at the end of each run, specificity of PCR products was checked by melting curve analysis and agarose gel electrophoresis**.** The probe signal was normalized to an internal reference, and a cycle threshold (Ct) was taken above the background fluorescence.

PCR conditions used to amplify samples for DNA pyrosequencing

PCR conditions were as follows: 1x buffer Platinum Taq (Invitrogen), 0.2 mM deoxynucleotides, 1.5 mM $MgCl_2$, 1U DNA polymerase Taq Platinum (Invitrogen), 0.5 µM primers forward and reverse and 4 ng of DNA template. PCR amplification included a starting denaturation at 94°C for 2 min, 40 PCR cycles of 94°C for 20 sec for denaturation, see supplementary Table 1 for annealing temperature and 72°C 1 min, followed by a final extension at 72°C for 7 min.

Quantitative reverse-transcription polymerase chain reaction (qRT-PCR)

QRT-PCR was carried out in 25 µl total volume containing 4 ng of cDNA, 1x Power SYBR Green I Master Mix (Applied Biosystems), 400 nM of each primer. After a starting denaturation for 10 minutes at 95°C, 45 PCR cycles (15s 95°C and 1 min 60°C) qRT-PCR have been performed on ABI PRISM 7900HT SDS instrument (Applied Biosystems). Each sample was evaluated in triplicate and at the end of each run, specificity of PCR products was checked by melting curve analysis and agarose gel electrophoresis**.** To exclude the presence of DNA as a contaminant of RNA, a no reverse transcriptase cDNA was also amplified as a control. The probe signal was normalized to an internal reference, and a cycle threshold (Ct) was taken above the background fluorescence.
