# Supplementary material for: Methylation-associated down-regulation of RASSF1A and up-regulation of RASSF1C in pancreatic endocrine tumors
Source: BMC Cancer. 2011 Aug 12;11:351. doi: 10.1186/1471-2407-11-351 (PMC3170651; doi:10.1186/1471-2407-11-351)
Supplement: Additional file 2 — Additional tables. Table S1. Oligonucleotides and experimental conditions used for quantitative MSP (qMSP), DNA pyrosequencing, microfluidic chip-electrophoretic separation (RT-PCR) and quantitative RT-PCR (qRT-PCR). The table lists the primers sequences and the PCR conditions used in qMSP, RT-PCR and qRT-PCR. Table S2. Expression and statistics data of RASSF1A and RASSF1C in PET and normal pancreas obtained by quantitative RT-PCR (qRT-PCR). The table provides the expression data of RASSF1A and RASSF1C and the statistical analysis. [file 1471-2407-11-351-S2.PDF]

Additional file 2- Additional Tables

Table S1. Oligonucleotides and experimental conditions used for quantitative MSP (qMSP), DNA pyrosequencing, microfluidic chip-electrophoretic separation (RT-PCR) and quantitative RT-PCR (qRT-PCR).

	**Oligonucleotide (5'-3')***	**Annealing (°C)**	**$MgCl_2$^ (mM)**	**PCR# product**
**qMSP**				
RASSF1A	F-GCGTTGAAGTCGGGGTTC<br>R-CCCGTACTTCGCTAACTTTAAACG<br>P: FAM-ACAAACGCGAACCGAACGAAACCA-TAMRA	66	2.5	74
MYOD1	F-CCAACTCCAAATCCCCTCTCTAT<br>R-TGATTAATTTAGATTGGGTTTAGAGAAGGA<br>P: FAM-TCCCTTCCTATTCCTAAATCCAACCTAAATACCTCC-TAMRA	66	2.5	110
**Pyrosequencing**				
RASSF1A, promoter	F-GGATTAGGAGGGTTAGGGT<br>R-CTCCCCCAAAATCCAAAC<br>S-TAAAGTTGGTTTTTAGAAAT	62	1.5	288
RASSF1A, first exon (A)	F-AGTTTGGATTTTGGGGGAGG<br>R-CAACTCAATAAACTCAAACTCCCC<br>S-GGGTTAGTTTTGTGGTTT	62	1.5	136
RASSF1A, first exon (B)	F-GACGGGACACCGCTGATCGTTTAGGGTTATGTYGGG<br>F-GGGACACCGCTGATCGTTTA<br>R-CCACRACCAAAAACCAACTAC<br>S-AACTACCRTATAAAATTACA	57	1.5	180
RASSF1A, first exon (C)	F-GGGAGTTTGAGTTTA<br>R-CACTACAAACCTTTACA<br>S-TGTAATTTTATAGGGTAGTT	57	1.5	238
RASSF1A, antisense strand	F-GGGTTGGAAGAGGTGGTTA<br>R-CCTACACCCAAATTTCCATT<br>S-TGGTTAAGGTCAGGGAT	62	1.5	326
RASSF1C, promoter	F-TTGTGGTAGGTGGGGTTTGT<br>R-CCCAAATCTAACTCTTATCTCATTAAA<br>S-TGGGGTTTGTGAGTGGA	58	1.5	118
RASSF1C, first exon	F-TTTTGTTTTAATGAGATAAGAGTTAGA<br>R-AATCCTCTTAACTACAATAACCACTAC<br>S-TTTAATGAGATAAGAGTTAGAT	58	1.5	256
**RT-PCR**				
RASSF1A,D,E,F	F-GCGTCGTGCGCAAAGG<br>R-TGCTGTTGATCTGGGCATTG	59	2	229,241 (D/E), 118
RASSF1G	F-AAGGCCTGCAGTGCGC<br>R-GCTGGAGGGCACAGAGACAG	60	2	94
**qRT-PCR**				
RASSF1A	F-CTTGCTAGCGCCCAAAGC<br>R-CAGCTCCCGCAGCTCAAT	60	U	82
RASSF1B	F-GCCCAGGTGGCCAACAT<br>R-CGCGGCAGCGGTAGTG	60	U	73
RASSF1C	F-TTCACCGCGCGAACCT<br>R-TGATCTTCTGCTCAATCTCAGCTT	60	U	106
RPLPO	F-ACATGTTGCTGGCCAATAAGGT<br>R-CCTAAAGCCTGGAAAAAGGAGG	60	U	127

* F: forward; R: reverse; S: sequencing; P: probe

^ U: unknown

base pair

Table S2. Relative *RASSF1A* and *RASSF1C* expression in 13 matched PET and normal pancreas.

**Cases***	**Relative *RASSF1A* expression**	**Standard deviation**	***P* value^**	**Relative *RASSF1C* expression**	**Standard deviation**	***P* value^**
N3	0.75	0.20	<0.05	0.21	0.06	<0.05
T3	0.44	0.06		0.90	0.04	
N5	2.20	0.10	<0.05	0.14	0.30	<0.05
T5	0.45	0.03		4.20	0.09	
N6	2.61	0.14	<0.05	0.11	0.04	<0.05
T6	0.63	0.09		1.10	0.09	
N7	0.46	0.09	<0.05	0.27	0.20	<0.05
T7	0.17	0.10		1.20	0.10	
N8	0.60	0.06	<0.05	0.14	0.05	<0.05
T8	0.26	0.08		4.42	0.08	
N9	1.57	0.22	<0.05	4.81	0.06	<0.05
T9	11.87	0.02		15.43	0.02	
N10	0.31	0.10	<0.05	0.50	0.02	<0.05
T10	0.05	0.04		0.20	0.10	
N11	3.60	0.09	<0.05	0.59	0.04	<0.05
T11	0.50	0.20		1.57	0.07	
N12	0.90	0.15	<0.05	0.72	0.07	<0.05
T12	0.20	0.05		0.49	0.04	
N13	0.90	0.06	<0.05	0.44	0.09	<0.05
T13	0.22	0.06		3.26	0.04	
N14	0.74	0.09	n.s.	0.06	0.03	<0.05
T14	0.68	0.02		3.01	0.09	
N15	9.30	0.10	<0.05	1.13	0.10	<0.05
T15	0.30	0.02		2.66	0.04	
N20	2.30	0.18	<0.05	0.73	0.06	<0.05
T20	0.10	0.04		1.54	0.10	

* N=normal pancreas, T=PET , ^ *t-test*. *P* value refers to the comparison N/T in matched samples.
n.s.=not significant
