# Supplementary material for: Methylation-associated down-regulation of RASSF1A and up-regulation of RASSF1C in pancreatic endocrine tumors
Source: BMC Cancer. 2011 Aug 12;11:351. doi: 10.1186/1471-2407-11-351 (PMC3170651; doi:10.1186/1471-2407-11-351)
Supplement: Additional file 3 — Additional figures. Figure S1. Analysis of methylation of RASSF1A by methylation-specific PCR (MSP) and quantitative MSP (qMSP). The figure shows examples of MSP results and a graph representing data obtained by qMSP. Figure S2. Pearson's correlations (r) between expression of RASSF1A and the average methylation of single CpGs. The graph shows the Pearson's correlation values (r) between RASSF1A expression level and the average methylation level for each CpG of the CpG island A. [file 1471-2407-11-351-S3.PDF]

Additional file 3- Additional figures

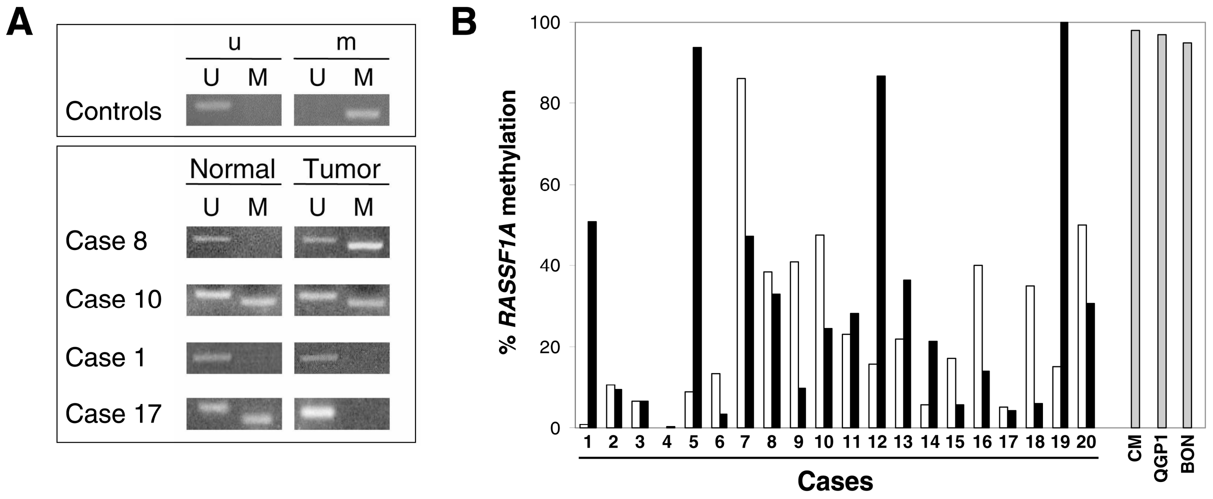

Figure S1. Analysis of methylation of *RASSF1A* by methylation-specific PCR (MSP) and quantitative MSP (qMSP). (A) Representative MSP results where PCR products from methylated (M) and unmethylated (U) samples are visualized on 3% agarose gels. Examples include: only tumor is methylated (case 8); both normal and tumor are methylated (case 10); both normal and tumor are unmethylated (case 1); only normal is methylated (case 17). Universal unmethylated DNA (u) and methylated DNA (m) were used as controls. (B) Results of qMSP in 20 paired PET/normal cases and three PET cell lines. Percent of methylation of *RASSF1A* was calculated by extrapolation from calibration curves, as described in Methods. 20 paired PET/normal cases are indicated with numbers from 1 to 20 as listed in Table 1. Black bar refers to PET, white bar refers to matched normal pancreas and grey bar refers to PET cell lines CM, QGP1, BON.

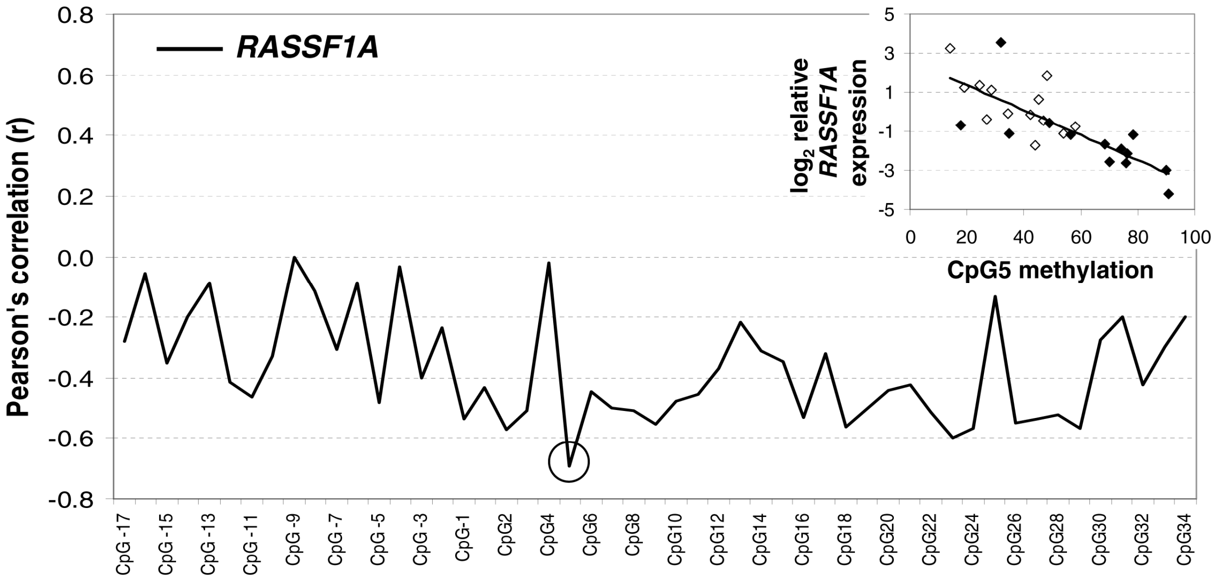

Figure S2. Pearson's correlations (r) between expression of *RASSF1A* and the average methylation of single CpGs. Variation of Pearson's correlation (r) of *RASSF1A* expression *vs* the average methylation of single CpGs in PET and normal pancreas. The circle highlights the r value at CpG5. The inner graph shows the relationship between the methylation levels of CpG5 and the respective expression level of *RASSF1A* in PET (black diamond) and normal pancreas (white diamond). A linear regression through the data ($y=-0.064x + 2.6453$, $R^2 = 0.6187$) describes the relationship between methylation and gene expression.
